# Efficient and rapid digestion of proteins with a dual-enzyme microreactor featuring 3-D pores formed by dopamine/polyethyleneimine/acrylamide-coated KIT-6 molecular sieve

**DOI:** 10.1038/s41598-024-65045-w

**Published:** 2024-07-08

**Authors:** Fang-Fang Yuan, Pei Wang, Xiao-Jie Han, Ting-Ting Qin, Xin Lu, Hai-Jiao Bai

**Affiliations:** Tianjin Institute for Drug Control, Tianjin, 300070 China

**Keywords:** Dual-enzyme immobilization, KIT-6, Polydopamine, Polyethylenimine, Protein digestion, Biological techniques, Materials science

## Abstract

The microreactor with two types of immobilized enzymes, exhibiting excellent orthogonal performance, represents an effective approach to counteract the reduced digestion efficiency resulting from the absence of a single enzyme cleavage site, thereby impacting protein identification. In this study, we developed a hydrophilic dual-enzyme microreactor characterized by rapid mass transfer and superior enzymatic activity. Initially, we selected KIT-6 molecular sieve as the carrier for the dual-IMER due to its three-dimensional network pore structure. Modification involved co-deposition of polyethyleneimine (PEI) and acrylamide (AM) as amine donors, along with dopamine to enhance material hydrophilicity. Remaining amino and double bond functional groups facilitated stepwise immobilization of trypsin and Glu-C. Digestion times for bovine serum albumin (BSA) and bovine hemoglobin (BHb) on the dual-IMER were significantly reduced compared to solution-based digestion (1 min vs. 36 h), resulting in improved sequence coverage (91.30% vs. 82.7% for BSA; 90.24% vs. 89.20% for BHb). Additionally, the dual-IMER demonstrated excellent durability, retaining 96.08% relative activity after 29 reuse cycles. Enhanced protein digestion efficiency can be attributed to several factors: (1) KIT-6’s large specific surface area, enabling higher enzyme loading capacity; (2) Its three-dimensional network pore structure, facilitating faster mass transfer and substance diffusion; (3) Orthogonality of trypsin and Glu-C enzyme cleavage sites; (4) The spatial effect introduced by the chain structure of PEI and glutaraldehyde’s spacing arm, reducing spatial hindrance and enhancing enzyme–substrate interactions; (5) Mild and stable enzyme immobilization. The KIT-6-based dual-IMER offers a promising technical tool for protein digestion, while the PDA/PEI/AM-KIT-6 platform holds potential for immobilizing other proteins or active substances.

## Introduction

The bottom-up strategy, relying on mass spectrometry, has emerged as the predominant approach in proteomics^1–4^. A pivotal step in this process involves the enzymatic digestion of proteins into peptides, wherein protein enzymes cleave peptide chains by recognizing specific sequences^5,6^. With the progression of proteomics research, there is a growing demand for enhanced enzymatic performance. A single type of protein enzyme is no longer adequate to meet these demands, prompting many applications to employ the simultaneous use of two or more protein enzymes^7–9^. In reality, various kinds of proteins possess intricate and diverse structures. By combining proteinases with various cleavage sites, the number of missing cleavages can be reduced, increasing the number of peptide segments with optimal lengths (8–20 amino acids are more favorable for ionization efficiency), improving sensitivity of mass spectrometry (MS) detection, and providing more peptide information to enhance the accuracy of protein identification. Trypsin stands out as the most commonly utilized protease due to its ability to generate peptides of optimal length, its distribution of gas-phase charge states, and its specificity, rendering it highly compatible with mass spectrometry detection. Additionally, other proteases such as Glu-C, Lys-C, and chymotrypsin find application in protein analysis^7,10–13^. Currently, extensive research substantiates the efficacy of employing multi-enzyme combination digestion^10,11^. Studies have demonstrated the advantageous orthogonality of trypsin/Glu-C, with their combined usage shown to enhance enzymatic digestion efficiency^12,14,15^. For instance, Bai et al. developed Graphene-oxide (GO)-based immobilized trypsin and endoproteinase Glu-C, which, when used in tandem, improved protein identification and sequence coverage^14^. Similarly, Liang et al. successfully identified purified ricin using a trypsin/Glu-C tandem digestion approach^15^.

Mesoporous molecular sieves offer significant advantages, including a large specific surface area, uniform pore size distribution, and nanometer confinement effect, making them pivotal in enzyme immobilization^16,17^. The KIT-6 molecular sieve, characterized by its unique three-dimensional (3-D) hexagonal structure, boasts a higher enzyme loading capacity due to its expansive specific surface area^18–20^. Moreover, its 3-D interconnected pore structure, in contrast to one-dimensional or two-dimensional channel mesoporous structures, facilitates enhanced mass transfer and diffusion rates. This enables rapid dispersion of enzymes and accelerated diffusion of reactants and products during the reaction process, rendering it an ideal carrier for immobilized enzymes.

Dopamine facilitates carrier modification through self-polymerization, thereby improving carrier material hydrophilicity and reducing non-specific protein/peptide adsorption^21^. In this study, dopamine was employed to modify the surface of the KIT-6 molecular sieve, while polyethyleneimine (PEI) and acrylamide (AM) were added simultaneously during the deposition process as amine donors. This inhibited the precipitation of polydopamine (PDA) aggregates and extended the time window for stable co-deposition^22–25^. The residual amino and double bond functional groups resulting from PEI and AM polymerization facilitate enzyme immobilization. PEI, a branched polymer copolymer with abundant amino groups, readily links enzymes using glutaraldehyde (GA) as a spacer. The chain structure of PEI and GA extends the connected enzyme, reducing spatial hindrance^26^. Additionally, residual double bonds react with thiol groups on proteins, facilitating rapid and gentle immobilization of another enzyme without affecting the activity of the first immobilized enzyme^27,28^. Introduction of these functional groups in a single step enables stepwise immobilization of two enzymes, preventing cross-enzyme digestion; covalent grafting ensures IMER stability and durability. Furthermore, the stretched chain structure spatially distributes the two fixed proteases in a staggered manner, minimizing steric hindrance and maintaining enzyme conformation stability.

**Figure 1:**
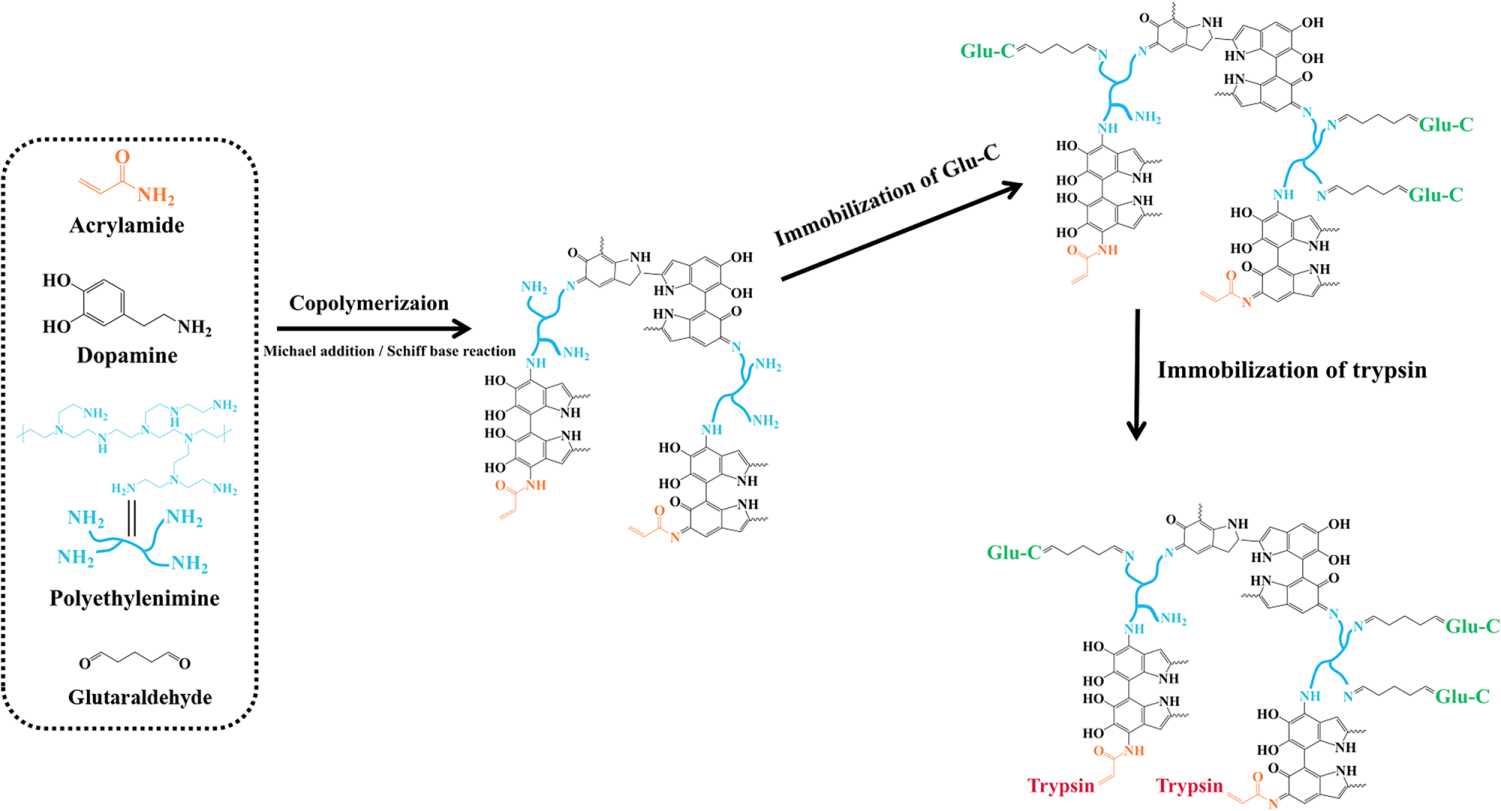
Scheme of the preparation of dual-IMER based on KIT-6.

This report introduces a novel dual-enzyme microreactor platform featuring enhanced mass transfer rates and spatially staggered enzyme distribution (Fig. 1). To design this immobilized enzyme carrier, (1) KIT-6 was selected for its 3-D interconnecting pore structure facilitating enzyme dispersion and substrate mass transfer; (2) Dopamine self-polymerization modified the carrier surface, while PEI and AM were introduced using a one-step method, with GA serving as the spacer arm. The steric hindrance of trypsin and Glu-C was mitigated by either Michael addition or Schiff base reaction to immobilize the two enzymes. Experimental investigations were conducted to determine optimal enzymolysis conditions, including buffer types, ionic concentrations, and enzymolysis time. The IMER's performance was evaluated by digesting standard proteins.

## Experimental

### Materials

Dithiothreitol (DTT), iodoacetamide (IAA), acetonitrile (ACN), trifluoroacetic acid (TFA), and formic acid (FA) were procured from Sigma-Aldrich (St. Louis, MO, USA). Glu-C was obtained from MP Biomedicals Co., Ltd. (Shanghai, China), while trypsin, glutaraldehyde (GA), acrylamide (AM), dopamine (DA), polyethyleneimine (PEI, Mw = 600 Da), Tris(2-carboxyethyl)phosphine hydrochloride (TCEP), N,Nʹ-methylenbisacrylamide (MBA), ammonium persulfate (APS), ammonium bicarbonate (NH_4_HCO_3_), 4-(2-Hydroxyethyl)-1-piperazineethanesulfonic acid, sodium dihydrogen phosphate, Tris(hydroxymethyl)-aminomethane (Tris), disodium hydrogen phosphate, bovine serum albumin (BSA), bovine hemoglobin (BHb), and urea were sourced from Aladdin Co., Ltd. (Shanghai, China). Deionized water was generated using a Milli-Q IQ 7000 system from Millipore (Bedford, MA). Mesoporous molecular sieve KIT-6, with a pore size of 10 nm, was supplied by JCNANO Reagent Co., Ltd. (Nanjing, China).

### Instrument

A high-resolution mass spectrometer (Orbitrap Exploris 480, Thermo Fisher Scientific) was employed for sample solution analysis. An analytical balance (Mettler Toledo, XS205) and a digital thermostatic metal bath (Thermo Fisher Scientific) were utilized for sample weighing and temperature regulation.

### Selection of enzymes

Trypsin, being the most commonly used protease, cleaves amino acid sequences at /k/r-\p sites, targeting the C-terminal of arginine and lysine residues^13,29,30^. However, for proteins lacking these residues, such as membrane proteins or those with high molecular weight, digestion efficiency tends to be lower. To enhance protein digestion efficiency, it's crucial to select two proteases with complementary cleavage sites. Glu-C (V8 protease), belonging to the serine protease family, hydrolyzes carboxy-side peptide bonds of glutamic acid (E) or aspartic acid (D) residues. Its cleavage specificity differs from trypsin, yielding distinct peptide segments that may offer additional identification sites^31,32^. Apart from complementary cleavage sites, the enzymolysis conditions of both enzymes should be similar, ensuring stability and high activity. Glu-C protease exhibits optimal enzymatic performance at 37 ℃ within the pH range of 8.0–8.5, selectively cleaving the carboxyl group of Glu in 0.01–0.1 mol/L NH_4_HCO_3_ buffer (pH7.8). Simultaneous cleavage of the carboxyl groups of Glu and Asp occurs in 0.01–0.1 mol/L phosphate buffer (pH7.8). Similarly, trypsin demonstrates high enzyme activity in various buffers with a pH around 8.0 at 37 ℃, including ammonium bicarbonate, phosphate buffer, and Tris–HCl buffer. Therefore, trypsin and Glu-C have similar enzymatic conditions, and it is promising to be fixed in the same carrier for synergistic proteolysis, potentially enhancing digestion efficiency and protein recognition coverage.

### Preparation of PDA/PEI/AM-KIT-6

The dopamine/polyethyleneimine/acrylamide-functionalized KIT-6 molecular sieve (PDA/PEI/AM-KIT-6) was prepared based on previous work with some modifications^33^. In brief, 50 mg of KIT-6 was added to 20 mL of Tris–HCl buffer (0.05 M, pH 8.5) and sonicated for 20 min. Subsequently, 40 mg of dopamine, 20 mg of acrylamide, and 20 mg of polyethyleneimine (PEI 600) were added and thoroughly mixed under magnetic stirring. After stirring for 12 h at 28 ℃, the mixture was centrifuged (10,000 rpm) for 10 min. The resulting solid was washed four times with deionized water through successive washing/centrifugation cycles at 10,000 rpm and then dried under reduced pressure at 40 ℃.

### Immobilization of Glu-C protease

To prevent cross-enzymolysis, a stepwise fixation method was employed for the immobilization of Glu-C on PDA/PEI/AM-KIT-6, based on previous work with some modifications^34^. Typically, PDA/PEI/AM-KIT-6 was dispersed in 2 mL of phosphate buffer (PBS, 50 mM, pH 6.0), followed by the addition of 1.0 mg of Glu-C and 5 μL of a 20 wt % aqueous solution of GA. After stirring for 24 h at 30 ℃, the solids were collected by centrifugation (10,000 rpm) for 10 min and washed several times with PBS to remove residual reactants. The resulting immobilized Glu-C was labeled as PDA/PEI/AM-KIT-6-Glu-C.

### Immobilization of trypsin

The protocols for trypsin immobilization on PDA/PEI/AM-KIT-6-Glu-C were consistent with previous work^35^. Briefly, TCEP (1 mg/mL) was added to Tris–HCl buffer (20 mmol/L, pH 8.0) containing trypsin (10 mg/mL) and reacted at 25 ℃ for 3 h to break the disulfide bonds. The reaction solution was then centrifuged at 4 ℃ for 10 min (10,000 rpm). MBA (2 mg/mL), APS (1 mg/mL), and PDA/PEI/AM-KIT-6-Glu-C-trypsin (1 mg/mL) were added to the supernatant and reacted at 4 ℃ for 2 h to fix trypsin.

### Protein sample preparation

Protein denaturation, disulfide reduction, and alkylation of standard proteins BSA or bovine hemoglobin (BHb) were performed according to previous protocols^35^. Following treatment, the samples were diluted with NH_4_HCO_3_ buffer (50 mmol/L, pH 8.0) and stored at − 20 ℃ for subsequent use.

### Digestion procedures

#### Digestion on dual-enzyme microreactor

Initially, 100 μL of denatured protein solution (1 mg/mL) was added to an EP tube containing 10 mg of PDA/PEI/AM-KIT-6-Glu-C-trypsin, and the mixture was gently shaken for 1 min at 37 ℃. Subsequently, the mixture was centrifuged at low temperature for 3 min (10,000 rpm), and the supernatant was subjected to LC–MS analysis.

#### Digestions in-solution

The in-solution enzymatic hydrolysis process proceeded as follows: The denatured protein solution used for dual-IMER was supplemented with trypsin at a ratio of 1:100 (w/w). Following overnight incubation in a thermostatic dry bath at 37 ℃, the sample solution underwent heat treatment at 100 ℃ for 5 min to terminate the reaction. Then, Glu-C protease (enzyme-to-substrate ratio 1:100, w/w) was introduced into the digestion solution and enzymatically hydrolyzed in a thermostatic dry bath at 37 ℃ for 18 h. Upon completion of enzymatic hydrolysis, the reaction was quenched by adding 1 μL of formic acid (FA). The digested fluid was stored at − 20 ℃ before further analysis.

### Reusability and reproducibility of dual-enzyme microreactor

To assess durability, the dual-enzyme microreactor was repeatedly exposed to BSA substrate, followed by enzyme activity assays to evaluate the reusability of PDA/PEI/AM-KIT-6-Glu-C-trypsin. After each use, 1 mL of NH4HCO3 buffer (50 mmol/L, pH 8.0) was added into the EP tube containing PDA/PEI/AM-KIT-6-Glu-C-trypsin, shook well for 30 min to desorbate the substrate. The enzyme reactor was then centrifuged, and the supernatant was discarded. This process was repeated three times to remove nonspecifically adsorbed digested peptides. The relative activity (%) was determined by comparing the sequence coverage achieved from digesting BSA in the Nth cycle with that of the first cycle.

### LC–MS/MS analysis and database searching

All sample solution tests were conducted on the Orbitrap Q-Exactive mass spectrometry system. The sample solution was gradient-eluted at a flow rate of 0.2 µL/min by an ultra-high-performance liquid chromatograph (UPLC) directly connected to the Orbitrap 480 Mass spectrometer (Thermo Fisher Scientific). Separations were performed on a C18 column (Waters, AQUITY BEH, 100 mm × 2.1 mm; 1.7 μm). The mobile phase consisted of 0.1% formic acid aqueous solution (phase A) and 0.1% formic acid acetonitrile solution (phase B). The Q-Exactive mass spectrometer operated in data-dependent mode, with a static MS spray voltage, an ion transfer tube temperature of 320 ℃, HCD collision energies of 27%, a scanning range of 200–2000 m/z, and a resolution of 60,000. The MS/MS data were analyzed using Xcalibur Foundation 3.0 SP2 (Thermo Fisher Scientific) and BioPharma Finder (Thermo Fisher Scientific).

### Ethical approval

This research did not involve human or animal samples.

## Results and discussion

### Characterization of KIT-6 before and after modification

**Figure 2:**
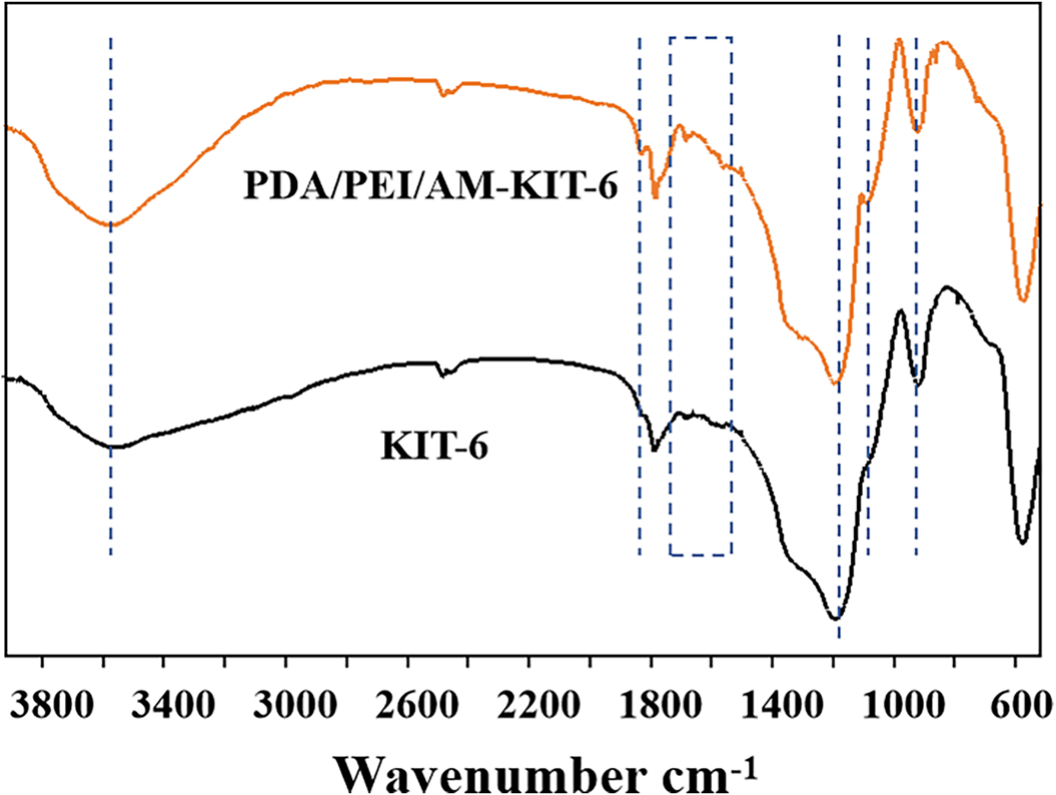
The FT-IR spectra of KIT-6 and PDA/PEI/AM-KIT-6.

Fourier Transform Infrared Spectroscopy (FTIR) was used to confirm the successful polymerization of dopamine, polyethyleneimine, and acrylamide on the surface of KIT-6. As shown in Fig. 2, KIT-6 and PDA/PEI/AM-KIT-6 both exhibited characteristic absorption peaks at 1186 cm^−1^ and 902 cm^−1^, attributed to the asymmetric stretching vibration of Si–O–Si and the stretching vibration of Si–O in the skeleton structure of KIT-6 molecular sieve. The results show that KIT-6 still has a complete structure after PDA/PEI/AM co-deposition on the surface. In the original KIT-6, an absorption peak can be observed at 3580 cm^-1^, which is caused by the stretching vibration of Si–O–H. In comparison to KIT-6, the peak intensity at 3581 cm^-1^ in PDA/PEI/AM-KIT-6 significantly increased due to the introduction of more O–H and N–H groups from PDA and PEI on the surface. The results showed that the surface of KIT-6 molecular sieve was successfully modified by PDA and PEI. Additionally, vibrations related to PDA result in the absorption peak at 800–900 cm^-1^ due to out-of-plane bending of the benzene ring; peaks at 1890 cm^-1^ correspond to the characteristic absorption peaks of C=O stretching vibration. After the deposition of PDA/PEI/AM, a new absorption band appeared at 1727 cm^-1^, corresponding to the stretching vibration peak of C=C. The spectral band at 1150 cm^-1^ was characteristic of =C–H bending vibration. However, the peak was not prominent at 1186 cm^-1^ due to the strong absorbance of Si–O–Si. Infrared results indicate that PEI, AM, and DA have been successfully co-deposited on the surface of KIT-6.

### Optimization of preparation parameters

#### Effect of the fixation order of two enzymes

**Figure 3:**
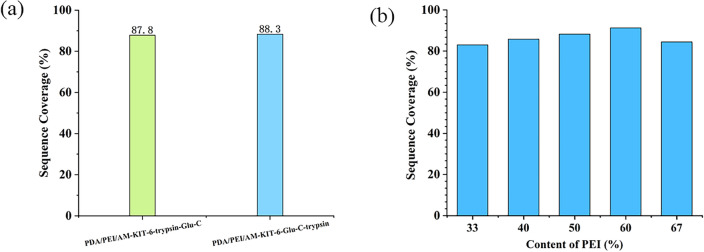
The effect of two enzyme fixation order (**a**) and ratio of PEI to AM (**b**). Digestion conditions: BSA concentration: 1 mg/mL in NH_4_HCO_3_ (pH 7.9, 50 mM); reaction temperature: 37 °C; reaction time: 1 min.

By incorporating polyethyleneimine (PEI) and acrylamide (AM) during dopamine self-polymerization, we facilitated the synthesis of polydopamine-polyethyleneimine-acrylamide copolymer on KIT-6's surface, introducing both amino and double bond functional groups in a single step. Reacting the aldehyde group of glutaraldehyde with the amino groups on the carrier surface and the amino groups on Glu-C to form a Schiff base, while trypsin, with cleaved disulfide bonds, was immobilized through thiol group reactions with double bonds. However, the order of enzyme immobilization is adjustable. To optimize enzymatic hydrolysis, we explored the fixed order of the two enzymes using BSA as the standard protein. The preparation method of PDA/PEI/ am-Kit-6-Trypsin-Glu-C was as follows: firstly, the trypsin was fixed on the surface of PDA/PEI/AM-KIT-6 according to “Immobilization of trypsin”, and then Glu-C was fixed according to “Immobilization of Glu-C protease”. Other procedures were the same as the preparation of PDA/PEI/AM-KIT-6-Glu-C-trypsin. The sequence coverage of the double-enzyme solution, PDA/PEI/AM-KIT-6-Glu-C-trypsin, and PDA/PEI/AM-KIT-6-trypsin-Glu-C is shown in Fig. 3a.

The dual-enzyme reactor, with Glu-C fixed first and then trypsin, exhibited a higher sequence coverage rate (88.30%), while the one with trypsin fixed first and then Glu-C had a lower sequence coverage rate (84.70%) compared to the double enzyme solution method (87.80%). This disparity may stem from the longer time needed to fix Glu-C via glutaraldehyde, potentially diminishing trypsin's activity during this process. Conversely, trypsin fixation conditions are more favorable, with shorter reaction times and lower temperatures conducive to preserving enzyme activity. These results suggest that PDA/PEI/AM-KIT-6-Glu-C-trypsin achieves more effective enzymatic hydrolysis.

#### Effect of ratio of polyvinylimide to acrylamide

In our study, we prepared dual-enzyme reactors with varying PEI to AM ratios (the PEI content is from 33 to 67%) to investigate their effects on enzyme activity. Figure 3b illustrates that as the proportion of PEI increases, sequence coverage shows an upward trend. At a PEI ratio of 60%, the dual-enzyme reactor exhibits optimal enzymatic performance. However, exceeding 60% PEI proportion resulted in a declining trend in sequence coverage. This trend might arise from PEI's ability to enhance dopamine deposition uniformity and density, thereby improving KIT-6 molecular sieve mass transfer efficiency. Moreover, PEI and AM involvement in dopamine deposition and polymerization, through Michael addition or Schiff base reactions, introduces amino and double bond functional groups. Their ratio may influence reaction group quantity, thereby affecting enzyme immobilization and reactor activity.

### Optimization of enzymatic hydrolysis conditions

Selecting multiple enzymes with orthogonality for co-enzyme digestion can significantly enhance enzymatic hydrolysis efficiency. However, to achieve effective co-digestion of multiple proteases, it's crucial to identify appropriate digestion conditions to fully utilize their cleavage performance and synergistic effects. In this study, we investigated key enzymolysis conditions, including buffer types and concentrations, to determine the most suitable enzymolysis conditions.

**Figure 4:**
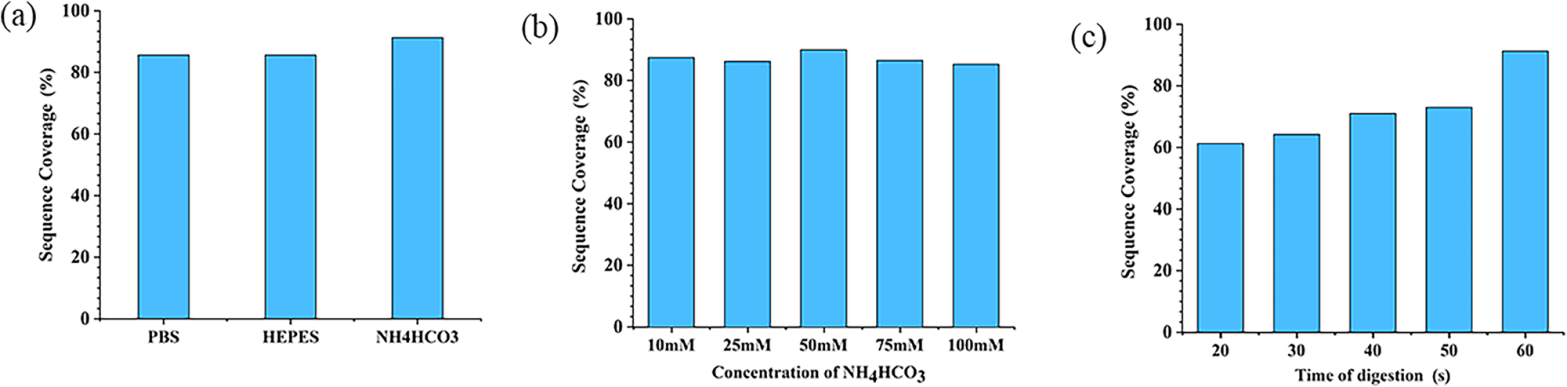
Effects of buffer salt type (**a**), concentration (**b**) and digestion time (**c**) on trypsin activity. Preparation Parameters of Dual-IMER: the content of PEI was 60%; BSA concentration: 1 mg/mL; reaction temperature: 37 °C; reaction time: 1 min.

Buffers play a crucial role in maintaining solution pH and preserving the integrity of biological macromolecules, thereby enhancing enzyme activity and stability. Phosphate buffer (PBS), 4-(2-Hydroxyethyl)-1-Piperazineethanesulfonic acid buffer (HEPES), and ammonium bicarbonate buffer (NH_4_HCO_3_) were identified as suitable for enzymatic hydrolysis experiments with Glu-C and trypsin. The enzymatic performance of the dual-enzyme reactor was evaluated under these conditions. As depicted in Fig. 4a, the dual-enzyme microreactor exhibited optimal enzymatic performance in NH_4_HCO_3_.

The ion concentration of the buffer solution can influence enzymatic hydrolysis efficiency by affecting enzyme–substrate interactions. Hence, we examined the impact of NH_4_HCO_3_ ion concentration ranging from 10 to 100 mM. As illustrated in Fig. 4b, a concentration of 50 mM ammonium bicarbonate was found to be the most suitable.

To determine the optimal enzymatic digestion time, dual-IMER conducted enzymatic digestion experiments at 37 ℃ under shaking conditions for various durations. As observed in Fig. 4c, with increasing enzymatic hydrolysis time, sequence coverage gradually increased. Notably, when the hydrolysis time reached 60 s, dual-IMER exhibited the best enzymatic hydrolysis effect, with a sequence coverage of 91.3%. Proteins, being large molecules with numerous enzyme cleavage sites, require adequate contact time with enzymes. Insufficient contact time may lead to incomplete cleavage of protein cutting sites. The dual-enzyme reactor developed in this study accomplished the protein digestion process in just 1 min.

### Reusability and reproducibility test of dual-IMER

**Figure 5:**
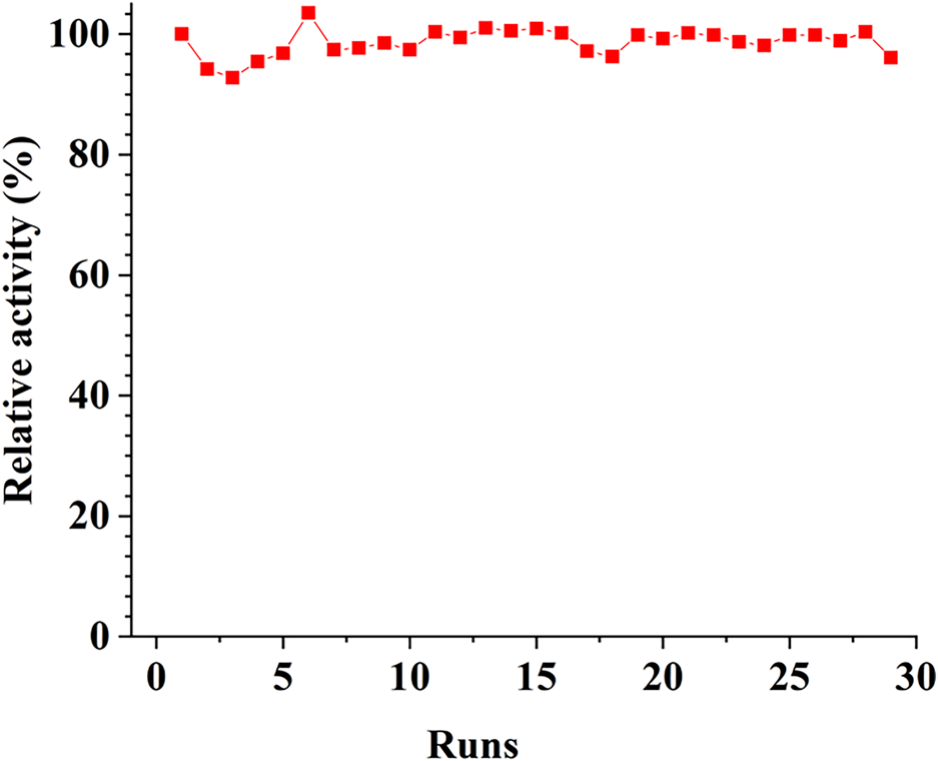
Reusability of dual-IMER. The ratio of sequence coverage between the Nth cycle and the 1st cycle was used to evaluate relative activity (%). Conditions was same as Fig. 4.

Reusable enzyme reactors offer benefits in reducing production costs and promoting sustainable development. In this experiment, the reusability of the dual-enzyme reactor was assessed by repeatedly using the same IMER to enzymatically hydrolyze the BSA substrate. As depicted in Fig. 5, after 29 cycles, the enzyme reactor's relative activity did not significantly decrease, with a range of 92.75–103.51%. Table 1 compares the reusability of immobilized enzyme reactors. The IMER prepared in this study demonstrated a higher number of recyclable uses compared to literature. This enhanced durability may be attributed to the covalent grafting method of enzymes and reduced mass transfer resistance due to the three-dimensional pore structure of KIT-6 molecular sieves.

**Table 1:** Comparison of digestion results of different material-based dual-IMERs for protein.

Material	Enzyme	Immobilization	Substrates	Sequence coverage (%)	Reusability	Refs.
SBA-15 hybrid monolithic column	Trypsin/chymotrypsin	Covalently fixed to two carrier	Carbonic anhydrase	88.5	5 cycles	^9^
Capillary monolithic support	Trypsin and chymotrypsin	Covalently fixed to one carrier	TRF	41.8–56.4	–	^11^
SBA-15 hybrid monolithic column	Trypsin or chymotrypsin	Covalently fixed to two carrier	BSA	81.7	–	^12^
GO-Trypsin and GO-Glu-C	Glu-C; trypsin	Covalently fixed to two carrier	BSA	90; 77	–	^14^
PDA/PEI/AM-KIT-6-Glu-C-trypsin	Glu-C and trypsin	Covalently fixed to one carrier	BSA	91.3	29 cycles	This work

Additionally, an ideal immobilized enzyme reactor should exhibit good intra- and inter-batch precision. The intra-batch (n = 3) and inter-batch (n = 4) precision of the dual-IMER prepared in this study were 0.6% and 0.9% (refer to Table 2), respectively. This increased durability may be attributed to the synergistic action of two enzymes and enzyme fixation through covalent bonding. In conclusion, PDA/PEI/AM-KIT-6-Glu-C-trypsin demonstrates good reusability and reproducibility.

**Table 2:** The intra-batch and inter-batch precision of the dual-IMER. Digestion conditions was same as Fig. 4.

	Intra-batch (n = 5)		Inter-batch (n = 3)	
	Sequence coverage (%)	RSD (%)	Recovery (%)	RSD (%)
BSA	86.16	0.6	87.97	0.9

### Comparison of dual-IMER with in-solution digestion

Enzymatic hydrolysis reactions pose greater challenges for larger molecular weight proteins due to their increased amino acid count and complex three-dimensional structure. BSA, with a molecular weight of 66.5 KDa and 583 amino acid residues containing 17 disulfide bonds, and BHb, a protein with a specific helical structure weighing 64.5 KDa, composed of four subunits each containing a hemoglobin molecule and an iron atom, were chosen for investigation. To explore the practical application of dual-IMER, the digestion efficiency of BSA and BHb by both the dual-enzyme microreactor and solution method was evaluated. Enzymatic hydrolysis procedures followed those outlined in “Digestion procedures”. For BSA and BHb, the sequence coverage identified by in-solution digestion (32 h) was 76.90% and 89.20%, respectively (refer to Table 3). The sequence coverage identified by the dual-IMER method (1 min) was 91.30% and 90.24%, respectively.

**Table 3:** Comparison of digestion results of two digestion methods for BSA and BHb. Digestion conditions: BSA concentration: 1 mg/mL in NH_4_HCO_3_ (pH 7.9, 50 mM); reaction temperature: 37 °C; reaction time: 1 min.

Digestion methods	Sequence coverage (%)	
	BSA	BHb
In-solution	76.90	89.20
PDA/PEI/AM-KIT-6-Glu-C-trypsin	91.30	90.24

The results demonstrate that dual-IMER achieve the same enzymatic digestion effect as traditional in-solution digestion but in only a fraction of the time, with higher sequence coverage. This efficiency improvement may be attributed to the longer spacer arm obtained by coupling PEI and GA, creating spatial distance for Glu-C to directly immobilize trypsin through reaction with thiol groups, thus reducing steric hindrance and potentially enhancing enzyme–substrate interaction. Furthermore, the unique three-dimensional porous structure of the KIT-6 carrier provides higher enzyme loading capacity and facilitates substance diffusion and mass transfer, preventing enzyme aggregation and loss of activity while allowing rapid access of large molecular substrates to the catalytic site. In order to ensure equal protein amounts between the two digestion methods, the solution's digestive fluid was diluted tenfold to match the concentration achieved by dual-IMER digestion.

## Conclusion

This study successfully prepared a dual-enzyme microreactor based on KIT-6 as the carrier and applied it to protein sample digestion. During the co-deposition process, the optimal mass ratio of PEI to AM was determined to be 3:2. Furthermore, optimal digestion conditions were achieved by optimizing buffer type, concentration, and reaction time. The resulting dual-IMER exhibited high activity and durability owing to the hydrophilicity conferred by dopamine deposition, rapid mass transfer through 3-D pores, and excellent stability from enzyme covalent grafting. In summary, PDA/PEI/AM-KIT-6-Glu-C-trypsin can effectively hydrolyze complex proteins. Moreover, this work presents a simple and efficient strategy for preparing carrier-coupled platforms. The PDA/PEI/AM-KIT-6 platform can be used not only for protease immobilization but also for immobilizing any substance containing groups capable of reacting with aldehyde and double bonds, enabling the production of diverse biosensors. For instance, anchored alcohol dehydrogenase (ADH)- and lactate dehydrogenase (LDH) to simultaneously determine the acetaldehyde and pyruvate contents^36^; developed a dual enzyme system of glucose oxidase (GOx) and horseradish peroxidase (HRP) for glucose detection^37^.

## Data Availability

The data and materials generated during and/or analyzed during the current study are available from the corresponding authors on reasonable request.

## References

[1] Aebersold R, Mann M (2016). Mass-spectrometric exploration of proteome structure and function. Nature.

[2] de Godoy L, Olsen J, Cox J (2008). Comprehensive mass-spectrometry-based proteome quantification of haploid versus diploid yeast. Nature.

[3] Miller RM, Smith LM (2023). Overview and considerations in bottom-up proteomics. Analyst.

[4] Duong VA, Park JM, Lee H (2020). Review of three-dimensional liquid chromatography platforms for bottom-up proteomics. Int. J. Mol. Sci..

[5] Yu YQ, Gilar M, Lee PJ, Bouvier ES, Gebler JC (2003). Enzyme-friendly, mass spectrometry-compatible surfactant for in-solution enzymatic digestion of proteins. Anal. Chem..

[6] Vandermarliere E, Mueller M, Martens L (2013). Getting intimate with trypsin, the leading protease in proteomics. Mass. Spec. Rev..

[7] Glatter T, Ludwig C, Erik A, Aebersold R, Heck AJR, Schmidt A (2012). Large-scale quantitative assessment of different in-solution protein digestion protocols reveals superior cleavage efficiency of tandem lys-c/trypsin proteolysis over trypsin digestion. J. Proteome. Res..

[8] Cheng YH, Lee CH, Wang SY, Chou CY, Yang YJ, Kao CC, Wu HY, Dong Y, Hung WY, Su CY, Tseng ST, Tsai IL (2023). Multiplexed antibody glycosylation profiling using dual enzyme digestion and liquid chromatography-triple quadrupole mass spectrometry method. Mol. Cell. Proteomics.

[9] Shangguan L, Zhang L, Xiong Z, Ren J, Zhang R, Gao F, Zhang W (2015). Investigation of bi-enzymatic reactor based on hybrid monolith with nanoparticles embedded and its proteolytic characteristics. J. Chromatogr. A.

[10] Swaney DL, Wenger CD, Coon JJ (2010). Value of using multiple proteases for large-scale mass spectrometry-based proteomics. J. Proteome. Res..

[11] Meller K, Pomastowski P, Grzywiński D, Szumski M, Buszewski B (2016). Preparation and evaluation of dual-enzyme microreactor with co-immobilized trypsin and chymotrypsin. J. Chromatogr. A.

[12] Wang B, Shangguan L, Wang S, Zhang L, Zhang W, Liu F (2016). Preparation and application of immobilized enzymatic reactors for consecutive digestion with two enzymes. J. Chromatogr. A.

[13] Chen Q, Yan G, Zhang X (2015). Applying multiple proteases to direct digestion of hundred-scale cell samples for proteome analysis. Rapid Commun. Mass Spectrom..

[14] Bai HH, Pan YT, Ren XJ (2014). An ultra-fast and highly efficient multiple proteases digestion strategy using graphene-oxide-based immobilized protease reagents. Sci. China. Chem..

[15] Liang LH, Cheng X, Yu HL, Yang Y, Mu XH, Chen B, Li XS, Wu JN, Yan L, Liu CC, Liu SL (2021). Quantitative detection of ricin in beverages using trypsin/Glu-C tandem digestion coupled with ultra-high-pressure liquid chromatography-tandem mass spectrometry. Anal. Bioanal. Chem..

[16] Zhang Z, Zhang L, Zhang C, Zhang W (2014). Hybrid organic-inorganic monolithic enzymatic reactor with SBA-15 nanoparticles incorporated. Talanta.

[17] Yasutaka K, Takato Y, Takashi K, Kohsuke M, Hiromi Y (2011). Enhancement in adsorption and catalytic activity of enzymes immobilized on phosphorus- and calcium-modified MCM-41. J. Phys. Chem. B.

[18] Vinu A, Gokulakrishnan N, Balasubramanian VV, Alam S, Kapoor MP, Ariga K, Mori T (2008). Three-dimensional ultralarge-pore ia3d mesoporous silica with various pore diameters and their application in biomolecule immobilization. Chemistry.

[19] Yuan LY, Zhu L, Xiao CL, Wu QY, Zhang N, Yu JP, Chai ZF, Shi WQ (2017). Large-pore 3D cubic mesoporous (KIT-6) hybrid bearing a hard-soft donor combined ligand for enhancing U(VI) capture: An experimental and theoretical investigation. ACS Appl. Mater. Interfaces..

[20] Chai K, Yang X, Shen R, Chen J, Su W, Su A (2023). A high activity mesoporous Pt@KIT-6 nanocomposite for selective hydrogenation of halogenated nitroarenes in a continuous-flow microreactor. Nanoscale. Adv..

[21] Yao AR, Yan YQ, Tan L, Shi YD, Zhou M, Zhang Y, Zhu PX, Huang SJ (2021). Improvement of filtration and antifouling performance of cellulose acetate membrane reinforced by dopamine modified cellulose nanocrystals. J. Membrane Sci..

[22] Lv Y, Yang SJ, Du Y, Yang HC, Xu ZK (2018). Co-deposition kinetics of polydopamine/polyethyleneimine coatings:effects of solution composition and substrate surface. Langmuir..

[23] Zhang R, Su Y, Zhao X, Li Y, Zhao J, Jiang Z (2014). A novel positively charged composite nanofiltration membrane prepared by bio-inspired adhesion of polydopamine and surface grafting of poly(ethylene imine). J. Membr. Sci..

[24] Li MM, Xu J, Chang CY, Feng CC, Zhang LL, Tang YY, Gao CJ (2014). Bioinspired fabrication of composite nanofiltration membrane based on the formation of DA/PEI layer followed by cross-linking. J. Membr. Sci..

[25] Yang HC, Liao KJ, Huang H, Wu QY, Wan LS, Xu ZK (2014). Mussel-inspired modification of a polymer membrane for ultra-high water permeability and oil-in-water emulsion separation. J. Mater. Chem. A.

[26] Volokitina MV, Bobrov KS, Piens K, Eneyskaya EV, Tennikova TB, Vlakh EG, Kulminskaya AA (2014). Xylan degradation improved by a combination of monolithic columns bearing immobilized recombinant β-xylosidase from Aspergillus awamori X-100 and Grindamyl H121 β-xylanase. Biotechnol. J..

[27] Fan PR, Zhao X, Wei ZH, Huang YP, Liu ZS (2020). Robust immobilized enzyme reactor based on trimethylolpropane trimethacrylate organic monolithic matrix through “thiol-ene” click reaction. Eur. Polym. J..

[28] Kim HW, Lee HD, Jang SJ, Park HB (2015). Highly chlorine and oily fouling tolerant membrane surface modifications by in situ polymerization of dopamine and poly(ethylene glycol) diacrylate for water treatment. J. Appl. Polym. Sci..

[29] Korecká L, Jankovicová B, Krenková J, Hernychová L, Slováková M, Le-Nell A, Chmelík J, Foret F, Viovy JL, Bílková Z (2008). Bioaffinity magnetic reactor for peptide digestion followed by analysis using bottom-up shotgun proteomics strategy. J. Sep. Sci..

[30] Fischer F, Poetsch A (2006). Protein cleavage strategies for an improved analysis of the membrane proteome. Proteome Sci..

[31] Chen R, Jiang X, Sun D, Han G, Wang F, Ye M, Wang L, Zou H (2009). Glycoproteomics analysis of human liver tissue bycombination of multiple enzyme digestion and hydrazide chemistry. J. Proteome Res..

[32] Schlosser A, Vanselow JT, Kramer A (2005). Mapping ofphosphorylation sites by a multi-protease approach with specific phosphopeptide enrichment and NanoLC-MS/MS analysis. Anal. Chem..

[33] Zhai TT, Wang CH, Gu FJ, Meng ZH, Liu WF, Wang YZ (2020). Dopamine/polyethylenimine-modified silica for enzyme immobilization and strengthening of enzymatic CO_2_ conversion. ACS Sustain. Chem. Eng..

[34] Sun J, Wang CH, Wang YZ, Ji SX, Liu WF (2019). Immobilization of carbonic anhydrase on polyethylenimine/dopamine codeposited membranes. J. Appl. Polym. Sci..

[35] Jiao YJ, Yuan FF, Fan PR, Wei ZH, Huang YP, Liu ZS (2020). Macroporous monolithic enzyme microreactor based on high internal phase emulsion functionalized with gold nanorods for enzymatic hydrolysis of protein. Chem. Eng. J..

[36] Shi J, Zhao W, Chen Y, Guo L, Yang L (2012). A replaceable dual-enzyme capillary microreactor using magnetic beads and its application for simultaneous detection of acetaldehyde and pyruvate. Electrophoresis.

[37] Juska VB, Pemble ME (2020). A dual-enzyme, micro-band array biosensor based on the electrodeposition of carbon nanotubes embedded in chitosan and nanostructured Au-foams on microfabricated gold band electrodes. Analyst.

